# Can European Countries Improve Sustainability of Health Care Financing through Patient Cost-Sharing?

**DOI:** 10.3389/fpubh.2015.00196

**Published:** 2015-08-12

**Authors:** Marzena Tambor, Milena Pavlova, Stanisława Golinowska, Wim Groot

**Affiliations:** ^1^Department of Health Economics and Social Security, Institute of Public Health, Faculty of Health Sciences, Jagiellonian University Collegium Medicum, Krakow, Poland; ^2^Department of Health Services Research, CAPHRI School for Public Health and Primary Care, Faculty of Health, Medicine and Life Sciences, Maastricht University Medical Center, Maastricht University, Maastricht, Netherlands; ^3^Top Institute for Evidence-Based Education Research (TIER), Maastricht University, Maastricht, Netherlands

**Keywords:** patient cost-sharing, Europe, health care financing, sustainable health care, value-based cost-sharing

## Introduction

Rising health care cost and resource constraints confront policy makers with the challenge to ensure the financial sustainability of health care systems, without jeopardizing the main health system objectives. To respond to this challenge, many European countries have introduced patient payments for publicly financed health care services (patient cost-sharing) (1–4). The potential of patient cost-sharing to contribute to the sustainability of the health care system relies on two elements. First, cost-sharing generates additional sources of funding. Hence, through cost-sharing, some of the health care cost might be shifted from public budgets to patients. Second, cost-sharing has the potential to improve efficiency in publicly financed health care, as it is expected that patients, when faced with the price of health care services, reduce the utilization of unnecessary and low-value health care (5, 6). It is also expected that this could slow the growth of health care costs. However, opponents of cost-sharing question the potential of cost-sharing to improve efficiency and instead point to its potentially negative effects on equity in health care. This is documented by evidence, among them the best known is the RAND health insurance experiment (7, 8).

Whether the potential of cost-sharing can be realized without threatening equity and consumers financial protection depends on various context-specific factors as well as on the design of the cost-sharing systems applied by European countries.

## Patient Cost-Sharing and Resources Generation

The ability of cost-sharing to generate revenues for the health sector is of particular interest to policy makers in countries with a poorly financed health care systems, where the lack of sufficient resources impedes the provision of health care services with an adequate quality and access. The evidence indicates that in many Central and Eastern European (CEE) countries, cost-sharing is seen primarily as a measure to reduce existing deficits in the underfinanced health care systems (9).

Designing a cost-sharing system, which raises substantial resources, is not a straightforward process and requires data on how consumers react to the change in the price of health care services. Higher charges might provide a greater potential for revenues. Yet, if demand is price sensitive, such cost-sharing system might substantially reduce the utilization of health care services. This limits the revenues generated, and also might adversely affect the population’s health status (if consumers forego the use of necessary care). For this reason, policy makers rather opt for cost-sharing to be sufficiently low to assure that the majority of consumers are able to pay the fees while offering even lower or no charges for those who cannot pay or who use health care frequently (10). A review of cost-sharing arrangements for health care services in European countries (1) shows that in CEE countries, co-payments (a flat fee) for out-patient visit ranges from approximately 1€ (Bulgaria, Czech Republic) to approximately 3€ (Latvia Estonia for a visit to specialist) (data for 2008–2009). In Western European countries, the fees are higher, yet their contribution to health care financing is still rather marginal, e.g., in Germany, a 10€ charge per first patient visit to the medical doctor in each calendar quarter, which existed till 2013, generated a net revenues of about two billion Euros a year (approximately 1% of public health insurance expenditure) (11). In some European countries (e.g., France, Slovenia), where cost-sharing takes the form of co-insurance and patients pay a percentage of health care cost, a private complementary health insurance, frequently purchased by consumers, takes over the responsibility and covers patients’ cost-sharing obligations.

The revenues from cost-sharing might be substantially restricted because of exemptions or compensations for selected population groups and payment limits, which are broadly applied in European countries. For example, in Latvia, due to exemptions (approximately one third of the population is exempted) and payments caps, the revenues from cost-sharing are reduced by half and accounted for 7% of total providers’ revenues (12). Although the presence of protection mechanisms, which are intended to diminish adverse equity effects of cost-sharing, deserves credit, their design, and applications leave much to be desired. The evidence indicates that the exemption/reduction mechanisms applied by European countries are not always well-targeted to those who need protection, for example, the criteria for the exemption/reduction includes occupation (e.g., medical professionals or war veterans are entitled) (13). In addition to an inadequate design of protection measures, their implementation sometimes fails in practice, for example, due to a problematic identification of vulnerable groups (e.g., low income individuals) or the complexity of the protection system, which is not transparent for patients and health care providers (14).

## Patient Cost-Sharing and Efficiency Improvements

In the well-funded health care systems of Western European countries, patient cost-sharing is often implemented as a measure to increase patient responsibility and thus, for a more efficient use of health care resources. Economic theory provides the rationale for the application of patient cost-sharing for the purpose of efficiency improvement. Since the price is a major determinant of the quantities of goods or services demanded, providing health care free-of-charge at the point of use (as it is in case of pure public financing) increases the quantity demanded (15–17). Part of this demand is considered to be excess demand since the marginal benefits of the consumption of these additional units of health care are lower than the marginal costs of their provision. From an economic point of view, efficiency then deteriorates as the best value for resources spent is not obtained (18). Thus, economic theory predicts that if consumers have to pay, they become more cost-conscious, i.e., they evaluate the expected benefits before the actual service use and utilize only those services whose benefits exceed the cost for them (5, 19). Imposing prices on the use of health care services is expected to affect also other forms of health-related behavior of health care consumers, i.e., provide incentives for a healthier lifestyle and prevention, which also might lead to efficiency gains in health care (20).

Nevertheless, the potential of cost-sharing to improve efficiency and further contain costs relies on the assumptions that the demand for health care is price sensitive and the decision on the use of services is made by consumers. Moreover, when making decisions, consumers are able to adequately value the services, i.e., estimate short- and long-term clinical benefits from the service consumption and the consequences of their behavior (21). While the first assumption is typically met, i.e., the quantity demanded for most health care services reacts to changes in price (the exemption can be, for example, lifesaving surgical procedures), the other assumptions are more doubtful (22).

First, the decision to use health care services is often not a patients’ choice but rather a physician’s decision and in such case implementing prices on services cannot be expected to change the quantity demanded. The evidence from the USA indicates that even if cost-sharing reduces the number of patient visits, the intensity of services provided remains unchanged, as it is largely driven by the providers (23). Therefore, cost-sharing alone without adequate supply-side measures, i.e., incentives for health care providers, has poor effectiveness in controlling the cost of health care (24). The importance of supply-side measures to improve efficiency and control cost of health care is well-recognized in Europe. Yet, more effort should be made to align demand- and supply-side measures for better performances of the health care systems.

Second, given the existing information issues (consumers’ insufficient medical knowledge, uncertainty), it is questionable whether consumers are able to adequately value the services and distinguish between low- and high-value services. Particularly, in case of services with positive externalities or merit goods (e.g., preventive services), it is well-recognized that individual or social benefits from their consumption are not fully recognized and considered by individual consumers (25). Hence, for a cost-sharing system to be able to enhance efficiency, it should give price signals to help consumers to discriminate between low- and high-value services. However, in European countries, the amounts of patient payments are generally not aligned with the values of the services for patients. Most countries apply uniform co-payments for broad categories of services (visit to a GP/specialists, hospitalization day) and few countries base the payment amounts on the actual service cost (co-insurance, deductibles) (1). Such “one size fits all” cost-sharing does not adequately moderate the utilization of services and is likely to reduce both essential and non-essential services, limiting the efficiency gain (26). This was confirmed in various studies, including the RAND Health Insurance Experiments conducted in the 1970s and more recent studies, which showed that an increase in patient cost-sharing results in the reduction of not only ineffective care but also of medically appropriate and essential care, and the low income and chronically sick are disproportionally affected by cost-sharing (7, 27–31). The evidence on the effects of cost-sharing policy in few European countries where such analyses have been performed, also confirms the adverse equity effects of cost-sharing (32–34). For example, the results of the study by Lostao et al. (32) indicate that patient cost-sharing in France reduces the frequency of physician visits and that this decrease is greater for persons from the lower socio-economic groups. Similarly, Rückert et al. (34) concluded that co-payments in Germany detained socially deprived patients from visiting a physician.

## A Step Forward

Despite the policy expectations for enhancing the sustainability of health care financing, the cost-sharing solutions applied by European countries have limited potential to improve efficiency or to generate additional resources. To better contribute to the sustainability of health care systems, cost-sharing arrangements in European countries should be reconsidered. The need to amend cost-sharing policies has been already put forward in health care debates. A new approach to cost-sharing called value-based cost-sharing (value-based insurance design) has been proposed (35). In this system, fees for health care services are differentiated based on their cost-effectiveness or on the health benefits they provide; health care services or goods, which are proven to be cost-effective are provided with no charges, particularly for patients who can benefit the most from their consumption (19, 36). Value-based cost-sharing has been increasingly applied for pharmaceuticals. In the area of health care services, it has been less common. Only some attempts to relate the level of fees to the value of services can be observed in European countries. For example, a review of cost-sharing arrangements for health care services in 27 EU countries (1) indicates that in a majority of these countries maternity and preventive services are excluded from the cost-sharing obligation. An interesting example comes from the Netherlands, where some insurers offer an option for their enrollees to be exempted from obligatory deductibles, if one uses services of preferred providers (i.e., providers who adhere to price and quality agreements) (37, 38). A common European countries’ practice of reducing fees for chronically ill should be also considered as a step toward value-based cost-sharing.

The main barrier to the implementation of value-based cost-sharing is the lack of data on the health benefits or cost-effectiveness of health care interventions and the high-administrative costs of such system. Nevertheless, European countries should consider a broader use of value-based cost-sharing in the future. This system could complement supply-side measures to improve quality and efficiency in health care, such as paying-for-performance and paying-for-coordination (39, 40). Furthermore, the fiscal efficiency of cost-sharing systems should be measured and should constitute important evaluation criteria of cost-sharing policy, particularly in countries, which aim to generate additional resources for health care through patient payments.

## Conflict of Interest Statement

The authors declare that the research was conducted in the absence of any commercial or financial relationships that could be construed as a potential conflict of interest.
